# Primary Glomus Tumor of the Inferior Vena Cava Diagnosed by Endovascular Biopsy: A Rare Incidental Finding

**DOI:** 10.7759/cureus.116728

**Published:** 2026-09-22

**Authors:** Bak Nin Choi Reina, Victor Carlo, Nickolle A Cruz Figueroa, Elba Torres Matundan, Rafael Mestres Franco

**Affiliations:** 1 Internal Medicine, Central Caribbean University, Bayamón, PRI; 2 Cytology, Puerto Rico Pathology, San Juan, PRI; 3 Pathology, Puerto Rico Pathology, San Juan, PRI; 4 Hematology and Oncology, Hospital Pavia Arecibo, Arecibo, PRI

**Keywords:** endovascular biopsy, glomus tumor, immunohistochemistry, inferior vena cava, intravascular tumor, vascular neoplasm

## Abstract

Glomus tumors are uncommon mesenchymal neoplasms with modified smooth muscle differentiation that classically arise in the subungual region but may occur at extradigital, visceral, and intravascular sites. Although primary intravascular glomus tumors have been reported, apparent primary origin within the inferior vena cava (IVC) represents an unusual presentation and must be distinguished from secondary caval involvement by an extravascular tumor.

A 44-year-old man with hypertension, diabetes mellitus, and osteoarthritis underwent musculoskeletal imaging for persistent right shoulder and upper back pain following a fall. Imaging incidentally demonstrated a 1.7-cm infrarenal IVC intraluminal abnormality initially interpreted as bland thrombus, while PET-CT showed no 18F-fluorodeoxyglucose (FDG)-avid malignancy. Endovascular evaluation demonstrated an obstructive intraluminal mass, and biopsy revealed uniform round-to-oval cells arranged around branching vascular channels. Immunohistochemistry demonstrated strong diffuse smooth muscle myosin heavy chain positivity with negative CAM 5.2 and chromogranin staining, supporting the diagnosis of glomus tumor. Subsequent surgical resection confirmed a 2.5-cm glomus tumor with smooth muscle actin (SMA) and caldesmon positivity and a low Ki-67 proliferative index (<1%).

This case demonstrates the diagnostic challenge of an indeterminate intraluminal IVC lesion and emphasizes the importance of distinguishing apparent primary caval origin from secondary IVC tumor involvement. Endovascular tissue sampling can provide a minimally invasive means of establishing a diagnosis when imaging findings are inconclusive, and the integration of imaging, histomorphology, immunohistochemistry, and surgical pathology is essential for definitive classification.

## Introduction

Glomus tumors are uncommon mesenchymal neoplasms showing modified smooth muscle differentiation and are classified within the pericytic (perivascular) family of tumors by the World Health Organization (WHO) [1,2]. They account for less than 2% of soft-tissue tumors and classically arise in the digits, particularly the subungual region, where they may present with localized pain, tenderness, and cold sensitivity [1]. Although historically regarded as predominantly digital tumors, contemporary institutional series have shown a substantial spectrum of extradigital disease, indicating that their anatomic distribution is broader than the classical subungual presentation [3].

Beyond their classical digital distribution, glomus tumors have been documented at numerous extradigital and visceral sites. The stomach represents one of the best-characterized visceral locations; a systematic review by Pansa et al. identified a total of 187 gastric glomus tumors across 89 studies, with 75.3% arising in the gastric antrum [4]. Intravascular glomus tumors are also an established presentation. Beham and Fletcher described a tumor arising from the wall of a forearm vein and protruding into its lumen [5], while Acebo et al. subsequently reported a 14-cm intravascular glomus tumor arising from a forearm vein [6]. Muneer et al. later summarized additional reported intravascular glomus tumors involving peripheral veins, particularly in the upper extremity [7]. These reports demonstrate that primary venous-wall origin is a recognized manifestation of glomus tumor and that vascular involvement itself is not unique to the present case.

It is important to consider that inferior vena cava (IVC) involvement by a glomus tumor must be distinguished from a tumor arising primarily within the caval wall or lumen. Secondary IVC involvement has been documented in association with extracaval glomus tumors. Alhouda et al. described metastatic disease from a previously resected gastric glomus tumor with tumor thrombus involving the IVC and extending to the IVC-right atrial junction [8]. More recently, Surianarayanan et al. reported a primary malignant renal glomus tumor with renal vein tumor thrombus extending into the IVC [9]. Together, these cases demonstrate that secondary IVC involvement can occur through venous tumor thrombus and should not be considered equivalent to primary caval origin. In contrast, our review of the available literature did not identify a previously reported glomus tumor clearly arising primarily from the IVC wall or lumen.

From a clinical and radiologic perspective, an intraluminal IVC mass also raises a reasonable consideration of bland thrombus, tumor thrombus from an extracaval malignancy, primary IVC neoplasms such as leiomyosarcoma, and intravenous leiomyomatosis [10-12]. Because imaging features may overlap among these entities, histopathologic evaluation with appropriate immunohistochemistry may be required for definitive classification [10].

This report describes a 44-year-old man with an incidentally identified 1.7-cm infrarenal IVC intraluminal abnormality initially interpreted radiologically as bland thrombus. Subsequent endovascular evaluation demonstrated an obstructive intraluminal mass, and endovascular biopsy established the diagnosis of glomus tumor, which was further characterized following surgical resection. This case highlights the diagnostic challenge posed by an indeterminate IVC lesion, the importance of distinguishing apparent primary caval origin from secondary IVC involvement, and the utility of endovascular tissue sampling when imaging alone cannot establish the diagnosis [13,14].

## Case presentation

A 44-year-old man with a medical history of hypertension, diabetes mellitus, and osteoarthritis presented with persistent right shoulder and upper back pain following a fall approximately one year prior. His shoulder symptoms prompted musculoskeletal imaging and were not initially attributed to an inferior vena cava (IVC) process. The vascular lesion was discovered incidentally during this diagnostic evaluation.

Imaging findings

MRI obtained during the evaluation of the patient's musculoskeletal complaint incidentally demonstrated an abnormality involving the inferior vena cava. Subsequent contrast-enhanced CT of the abdomen and pelvis, performed on October 11, 2025, and compared with a prior abdominal MRI from October 3, 2025, demonstrated a 1.7-cm intraluminal abnormality within the infrarenal IVC, radiologically interpreted as bland thrombus. PET-CT subsequently demonstrated no 18F-fluorodeoxyglucose (FDG)-avid malignancy. The patient underwent endovascular catheterization for presumed thrombectomy; however, intraprocedural fluoroscopy demonstrated an obstructive intraluminal mass rather than bland thrombus. Endovascular biopsy was therefore performed, yielding five fragments of tan soft tissue measuring 0.4 × 0.3 × 0.1 cm in aggregate. These measurements reflect the biopsy specimen, not the lesion's radiologic dimensions.

Histopathology

Microscopic examination revealed uniform round-to-oval cells with punched-out nuclei and eosinophilic cytoplasm, arranged in nests and sheets around branching thin-walled vascular channels (Figures 1, 2). The sampled tissue lacked necrosis, marked cytologic atypia, or brisk mitotic activity, supporting benign-appearing glomus tumor morphology.

**Figure 1:**
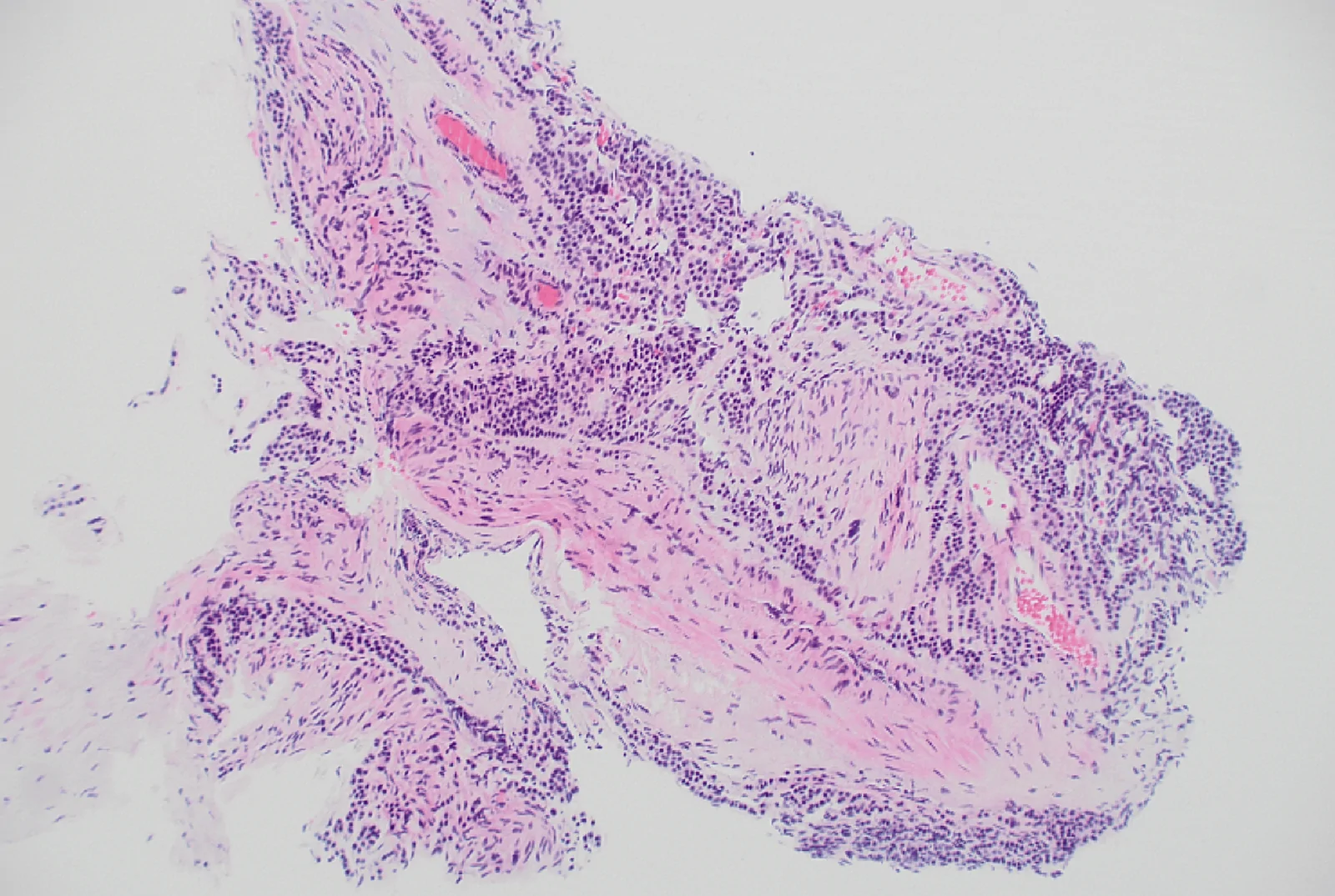
Histopathologic appearance of the inferior vena cava glomus tumor (H&E: ×100). Low-power hematoxylin and eosin (H&E) stain (×100) demonstrating a well-circumscribed proliferation of uniform round cells arranged in nests and sheets surrounding branching thin-walled vascular channels.

**Figure 2:**
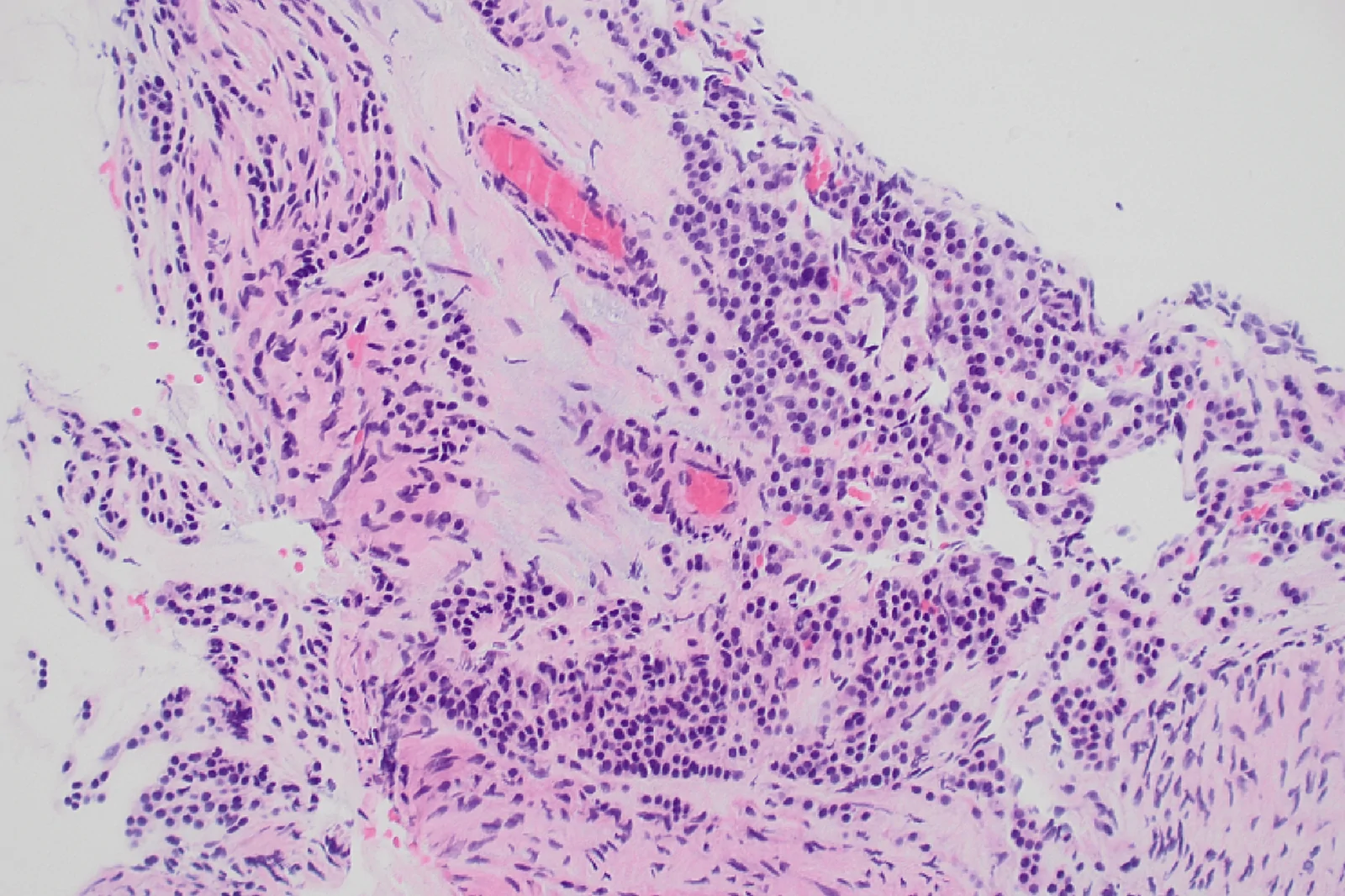
High-power histopathologic features of the glomus tumor (H&E: ×400). High-power hematoxylin and eosin (H&E) stain (×400) demonstrating uniform round cells with punched-out nuclei and eosinophilic cytoplasm, without marked cytologic atypia, necrosis, or readily identifiable mitotic activity, supporting benign-appearing histomorphologic features.

Immunohistochemistry

Immunohistochemistry was performed using a targeted panel consisting of smooth muscle myosin heavy chain, CAM 5.2, and chromogranin. Tumor cells demonstrated strong, diffuse cytoplasmic positivity for smooth muscle myosin heavy chain (Figure 3) and were negative for CAM 5.2 and chromogranin (Figures 4, 5). In conjunction with the characteristic histomorphologic features, this immunophenotypic profile supported the diagnosis of a glomus tumor of perivascular origin involving the inferior vena cava [2]. Table 1 summarizes the immunohistochemical findings.

**Figure 3:**
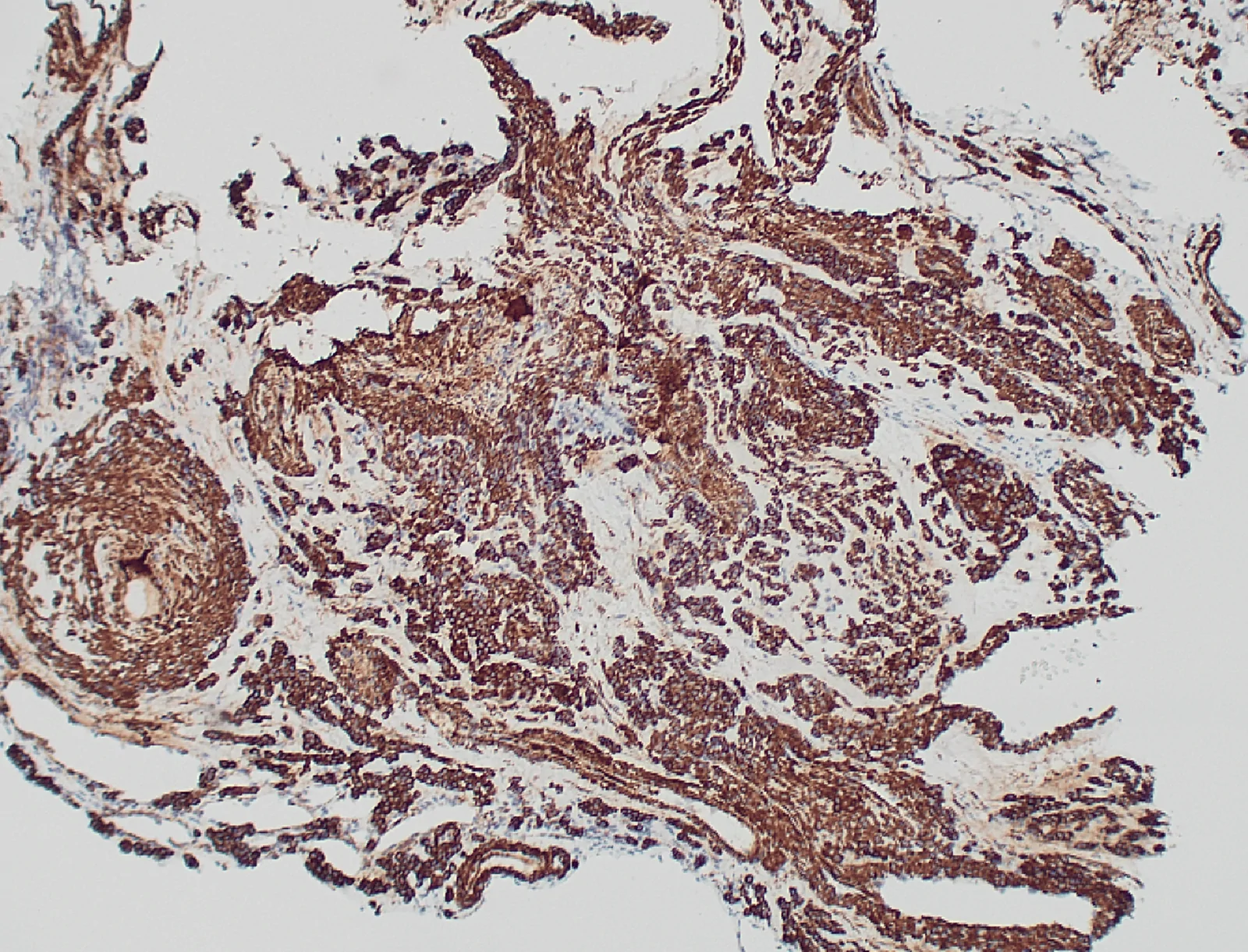
Diffuse smooth muscle myosin heavy chain positivity in glomus tumor cells. Immunohistochemical stain demonstrating strong, diffuse cytoplasmic positivity for smooth muscle myosin heavy chain in tumor cells, supporting smooth muscle/perivascular differentiation.

**Figure 4:**
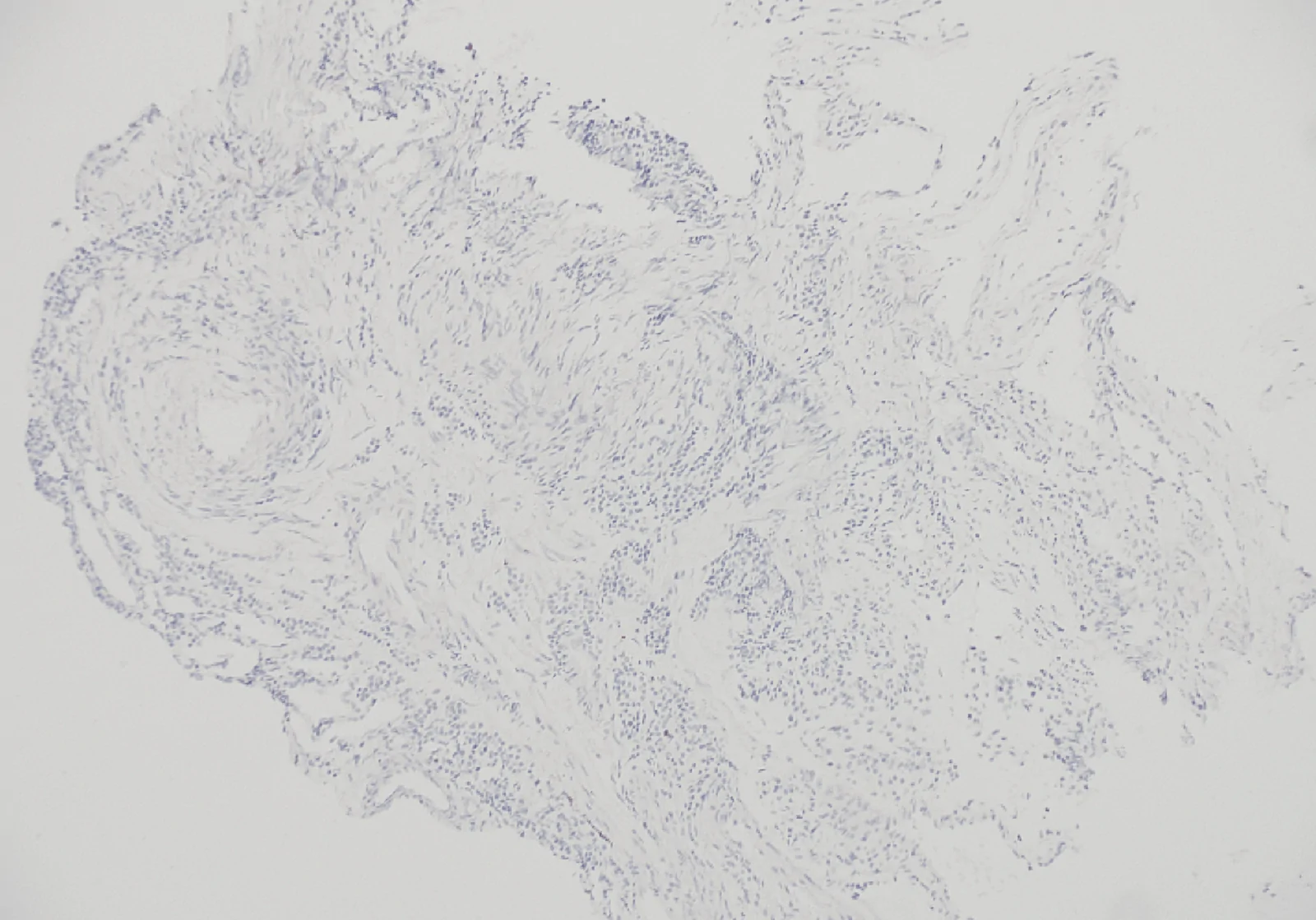
CAM 5.2 immunostain negative in tumor cells. Tumor cells show no CAM 5.2 immunoreactivity, arguing against epithelial differentiation.

**Figure 5:**
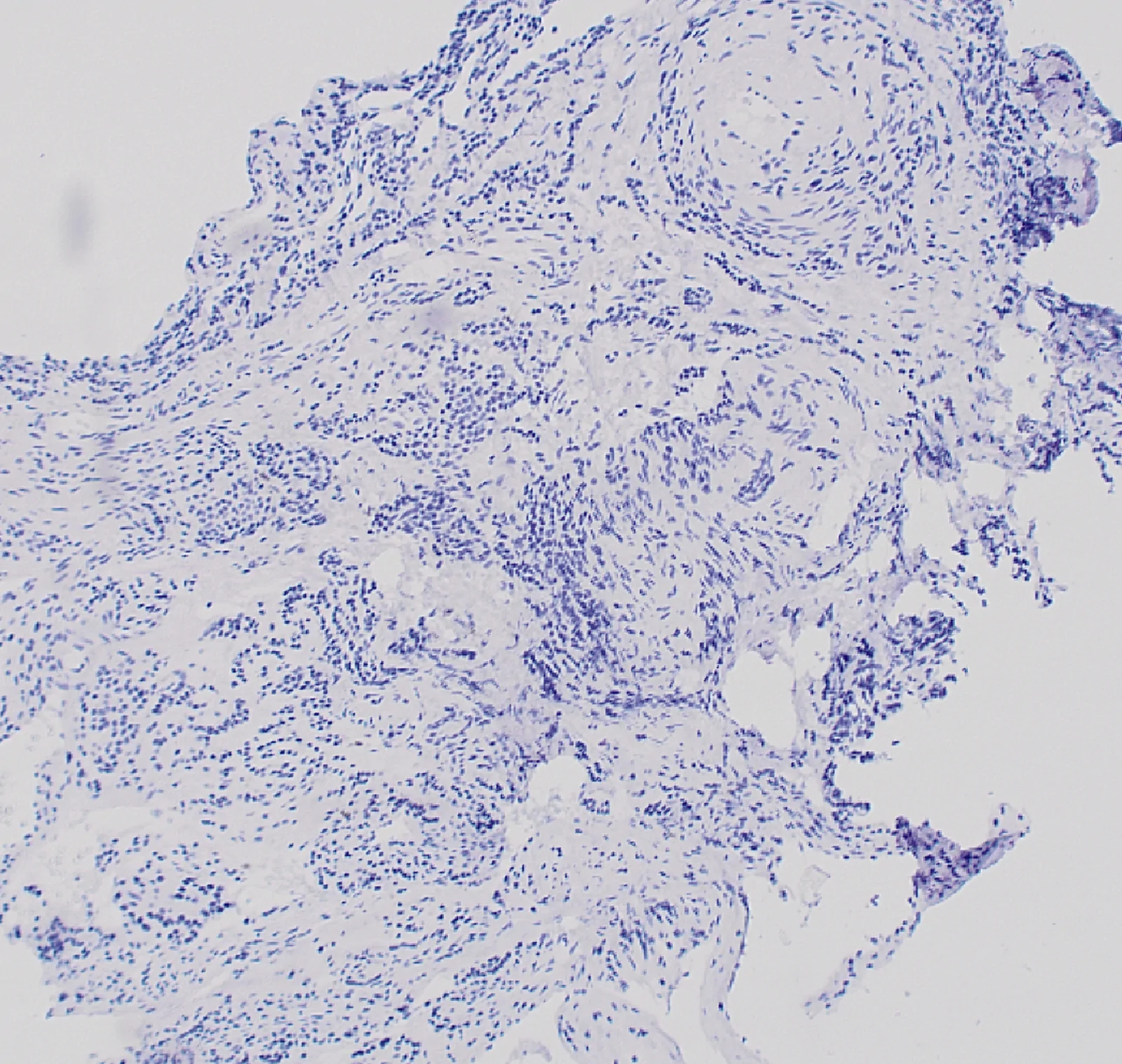
Chromogranin immunostain negative in tumor cells. Tumor cells are negative for chromogranin, arguing against a neuroendocrine neoplasm.

**Table 1:** Immunohistochemical profile of the inferior vena cava glomus tumor on endovascular biopsy and subsequent surgical resection. The initial endovascular biopsy was evaluated using a targeted immunohistochemical panel, whereas the subsequent surgical resection specimen underwent broader immunophenotypic characterization at a separate pathology laboratory. The table summarizes the diagnostically relevant findings from both specimens. SMA, smooth muscle actin; CAM 5.2, cytokeratin CAM 5.2; SOX10, SRY-box transcription factor 10

Specimen	Marker	Result	Diagnostic relevance
Endovascular biopsy	Smooth muscle myosin heavy chain (SMMHC)	Strong, diffuse positive	Supports smooth muscle/perivascular differentiation
Endovascular biopsy	CAM 5.2	Negative	Argues against epithelial differentiation
Endovascular biopsy	Chromogranin	Negative	Argues against neuroendocrine differentiation
Surgical resection	SMA	Positive	Supports smooth muscle/perivascular differentiation
Surgical resection	Caldesmon	Positive	Supports smooth muscle differentiation
Surgical resection	Desmin	Negative	Additional phenotypic characterization
Surgical resection	Synaptophysin	Negative	Argues against neuroendocrine differentiation
Surgical resection	Chromogranin A	Negative	Argues against neuroendocrine differentiation
Surgical resection	CD56	Partial positive	Additional phenotypic finding
Surgical resection	Ki-67	<1% proliferative index	Indicates low proliferative activity
Surgical resection	SOX10	Negative	Argues against neural/melanocytic differentiation

Surgical pathology

A separate pathology laboratory evaluated the surgical resection specimen obtained on June 8, 2026, and confirmed a 2.5-cm glomus tumor. Gross examination revealed a rubbery-to-firm soft-tissue mass measuring 2.5 × 1.5 × 1.3 cm and weighing 1.53 g. The cut surface was homogeneous and tan-brown without grossly identifiable necrosis.

The immunohistochemical evaluation of the surgical resection specimen demonstrated positivity for smooth muscle actin (SMA) and caldesmon, partial positivity for CD56, and a Ki-67 proliferative index of <1%. Tumor cells were negative for desmin, synaptophysin, chromogranin A, and SRY-box transcription factor 10 (SOX10). Additional stains were negative for CD45/LCA, OCT3/4, SALL4, CD117, CD30, INSM1, PAX8, CD38, and CD138. S-100, HMB-45, and Melan-A were not reported as having been performed. Table 1 summarizes the immunohistochemical findings from the initial endovascular biopsy and subsequent surgical resection.

Clinical course and outcome

Following histopathologic diagnosis, the patient subsequently underwent definitive surgical management on June 8, 2026, consisting of retroperitoneal exploration, the excision of the intracaval glomus tumor with the involved venous wall, and partial excision with the primary repair of the IVC wall. Postoperatively, he required intensive care monitoring and developed acute kidney injury, metabolic acidosis, hyperkalemia, pleural effusions, hyperglycemia requiring intravenous insulin, and hemodynamic instability requiring vasopressor support. His postoperative course was additionally characterized by hypovolemic shock and suspected reperfusion syndrome. Imaging demonstrated a large right pleural effusion and a small amount of postoperative hemoperitoneum. On June 11, 2026, the patient developed worsening hypotension with significant anemia and subsequently experienced cardiac arrest with asystole. Despite approximately 45 minutes of advanced cardiac life support, return of spontaneous circulation was not achieved, and he was pronounced deceased. The treating team documented the presumed cause of death as bleeding versus pulmonary embolism; a definitive cause was not established.

## Discussion

Glomus tumors classically arise in the distal extremities, particularly the digits and subungual region, but their anatomic distribution extends well beyond these conventional sites. Contemporary institutional series have documented a substantial spectrum of extradigital disease, while visceral tumors have been reported in several organs, with the stomach representing one of the best-characterized visceral locations [3,4]. Intravascular glomus tumors are also an established presentation. Beham and Fletcher [5] described a glomus tumor arising from the wall of a forearm vein and protruding into its lumen, and subsequent reports by Acebo et al. [6] and others documented additional primary intravascular tumors, predominantly involving peripheral veins [7]. These reports demonstrate that venous-wall origin itself is a recognized manifestation of glomus tumor. The unusual feature of the present case is therefore not vascular involvement alone but its apparent primary origin within the IVC.

Published IVC involvement by glomus tumor should also be classified by site of origin. Alhouda et al. described metastatic disease from a previously resected gastric glomus tumor with tumor thrombus involving the IVC and extending to the IVC-right atrial junction [8]. Surianarayanan et al. reported a malignant renal glomus tumor with renal vein tumor thrombus extending into the IVC [9]. These cases represent secondary caval involvement rather than a tumor arising primarily within the IVC. In contrast, our review of the available literature did not identify a previously reported glomus tumor clearly arising primarily from the IVC wall or lumen. This distinction provides a more specific basis for the unusual nature of the present case without directly implying that intravascular glomus tumors as a group are unprecedented.

From an imaging perspective, the differential diagnosis of an intraluminal IVC lesion includes bland thrombus, tumor thrombus from an extracaval malignancy, primary IVC neoplasms such as leiomyosarcoma, and intravenous leiomyomatosis. Features such as internal enhancement, vessel expansion, continuity with an underlying malignancy, and increased FDG uptake may favor tumor thrombus over bland thrombus, although imaging findings can overlap and are not invariably diagnostic [10]. Primary IVC leiomyosarcoma arises from the caval wall and may demonstrate intraluminal, extraluminal, or mixed growth, whereas intravenous leiomyomatosis typically demonstrates contiguous venous extension from a uterine or pelvic primary site and may extend through the IVC into the right-sided cardiac chambers [11,12]. In the present case, CT showed a 1.7-cm infrarenal IVC intraluminal abnormality initially interpreted as bland thrombus, while PET-CT showed no FDG-avid malignancy. Subsequent endovascular evaluation instead demonstrated an obstructive intraluminal mass, illustrating the potential limitations of imaging alone in characterizing an indeterminate caval lesion and the value of obtaining diagnostic tissue.

Histopathologically, the differential diagnosis includes smooth-muscle and other perivascular neoplasms, as well as neuroendocrine and epithelial mimics. Glomus tumors characteristically demonstrate relatively uniform round cells arranged around branching vascular channels and typically express smooth-muscle markers, including SMA and h-caldesmon [1-3]. Leiomyoma and leiomyosarcoma may share smooth-muscle marker expression but more typically demonstrate spindle-cell fascicular morphology; marked cytologic atypia, increased mitotic activity, and tumor necrosis further favor leiomyosarcoma in the appropriate morphologic setting. Accordingly, h-caldesmon expression supports smooth-muscle differentiation but does not independently distinguish glomus tumor from leiomyosarcoma. In the present case, the round-cell perivascular architecture, together with SMA and caldesmon positivity and the absence of desmin expression, supported glomus tumor over a conventional smooth-muscle neoplasm. Neuroendocrine tumors and paraganglioma may demonstrate nested architecture, but the absence of chromogranin and synaptophysin expression in the resection specimen, together with SOX10 negativity and the smooth-muscle phenotype, argued against these diagnoses. Perivascular epithelioid cell tumor (PEComa) may also show epithelioid and perivascular morphology with myoid differentiation but characteristically demonstrates melanocytic differentiation; HMB-45 and Melan-A were not reported as having been performed in this case. Metastatic carcinoma was considered less likely given the morphology and absence of CAM 5.2 immunoreactivity. Overall, the diagnosis was based on the integration of the characteristic morphology and immunophenotype rather than any single immunohistochemical marker.

The risk stratification of glomus tumors has evolved over time. Folpe et al. historically proposed criteria for malignant glomus tumor that included deep location and size greater than 2 cm, atypical mitotic figures, or a combination of moderate-to-high nuclear grade and increased mitotic activity [15]. Under these historical criteria, the deep location and 2.5-cm size of the present tumor would have represented concerning features. However, contemporary WHO classification no longer considers deep location and size greater than 2 cm alone sufficient for classification as malignant [2,16]. Current assessment places greater emphasis on morphologic features such as marked nuclear atypia and increased mitotic activity [2,16,17]. In the present case, the biopsy showed no marked cytologic atypia, tumor necrosis, or brisk mitotic activity, while the resection specimen demonstrated a low Ki-67 proliferative index (<1%) and no grossly identified necrosis. These findings support a low-proliferative phenotype, although the tumor's deep intravascular location and 2.5-cm size warrant the cautious interpretation of its biological potential.

Endovascular biopsy can provide a minimally invasive way to establish a tissue diagnosis in selected patients with indeterminate IVC lesions [13,14]. In the present case, endovascular evaluation transformed the working diagnosis from presumed thromboembolic disease to a perivascular neoplasm by demonstrating an obstructive intraluminal mass and permitting diagnostic tissue sampling. This distinction had direct clinical implications because the management strategy shifted from evaluating presumed bland thrombus to the definitive surgical treatment of an intracaval tumor. Surgical resection subsequently allowed the assessment of the entire 2.5-cm tumor and broader immunohistochemical characterization.

Definitive treatment consisted of the surgical excision of the intracaval tumor with the involved IVC wall, followed by partial resection and primary repair of the IVC. The postoperative course was complicated by acute kidney injury, high-anion-gap metabolic acidosis, hyperkalemia, pleural effusions, uncontrolled hyperglycemia, hemodynamic instability, and hypovolemic shock. On postoperative day 3, the patient developed worsening hypotension and anemia, followed by cardiac arrest, and died despite prolonged resuscitative efforts. The treating team documented bleeding versus pulmonary embolism as the presumed cause of death; a definitive cause was not established. Importantly, this fatal postoperative outcome should not be interpreted as evidence of the malignant biological behavior of the glomus tumor, as the death occurred in the immediate postoperative period in the setting of severe postoperative complications.

This case has several limitations. Although surgical resection permitted measurement of the 2.5-cm tumor and broader immunohistochemical characterization, long-term clinical follow-up was not possible because of the patient's postoperative death. The authors did not report a numerical mitotic count, limiting the more detailed assessment of proliferative activity, although the Ki-67 proliferative index was <1%. In addition, HMB-45 and Melan-A were not reported as having been performed, limiting the immunohistochemical assessment of melanocytic differentiation in the differential diagnosis with PEComa. Finally, the limited published literature specifically addressing apparent primary IVC glomus tumors restricts direct comparison with similar cases. Nevertheless, the combined radiologic, endovascular, histomorphologic, immunohistochemical, and surgical findings support the diagnosis of a glomus tumor involving the IVC, with findings favoring apparent primary caval origin.

## Conclusions

Glomus tumors may occur in intravascular locations, but apparent primary origin within the inferior vena cava represents an unusual presentation that must be distinguished from secondary caval involvement by an extracaval tumor. In this case, an incidental 1.7-cm infrarenal IVC abnormality initially interpreted as bland thrombus was subsequently recognized as an obstructive intraluminal mass and diagnosed as a glomus tumor by endovascular biopsy. Surgical resection confirmed the diagnosis and demonstrated a 2.5-cm tumor with smooth-muscle differentiation and a low Ki-67 proliferative index (<1%). Although the tumor showed benign-appearing histomorphology and low proliferative activity, its deep intravascular location and size warrant a cautious interpretation of its biological potential under contemporary glomus tumor classification. This case highlights the diagnostic value of endovascular tissue sampling for indeterminate intravascular lesions and emphasizes the importance of integrating imaging, histomorphology, immunohistochemistry, and surgical pathology when distinguishing primary caval neoplasms from bland thrombus and secondary tumor involvement.
